# Hijacking of Host Cellular Functions by an Intracellular Parasite, the Microsporidian *Anncaliia algerae*

**DOI:** 10.1371/journal.pone.0100791

**Published:** 2014-06-26

**Authors:** Johan Panek, Hicham El Alaoui, Anne Mone, Serge Urbach, Edith Demettre, Catherine Texier, Christine Brun, Andreas Zanzoni, Eric Peyretaillade, Nicolas Parisot, Emmanuelle Lerat, Pierre Peyret, Frederic Delbac, David G. Biron

**Affiliations:** 1 Clermont Université, Université Blaise Pascal, Laboratoire Microorganismes: Génome et Environnement, Clermont-Ferrand, France; 2 CNRS, UMR 6023, LMGE, Aubière, France; 3 Functional Proteomics Platform. UMR CNRS 5203, Montpellier, France; 4 Functional Proteomics Platform. UMS CNRS 3426, Montpellier, France; 5 INSERM, UMR1090 TAGC, Marseille, Marseille, France; 6 Aix-Marseille Université, UMR1090 TAGC, Marseille, France; 7 CNRS, Marseille, France; 8 Clermont Université, Université d'Auvergne, I.U.T., UFR Pharmacie, Clermont-Ferrand, France; 9 Clermont Université, Université d'Auvergne, EA 4678, Conception, Ingénierie et Développement de l'Aliment et du Médicament, Clermont-Ferrand, France; 10 Université de Lyon, Université Lyon 1, CNRS, UMR5558, Laboratoire de Biométrie et Biologie Evolutive, Villeurbanne, France; Duke University Medical Center, United States of America

## Abstract

Intracellular pathogens including bacteria, viruses and protozoa hijack host cell functions to access nutrients and to bypass cellular defenses and immune responses. These strategies have been acquired through selective pressure and allowed pathogens to reach an appropriate cellular niche for their survival and growth. To get new insights on how parasites hijack host cellular functions, we developed a SILAC (Stable Isotope Labeling by Amino Acids in Cell culture) quantitative proteomics workflow. Our study focused on deciphering the cross-talk in a host-parasite association, involving human foreskin fibroblasts (HFF) and the microsporidia *Anncaliia algerae*, a fungus related parasite with an obligate intracellular lifestyle and a strong host dependency. The host-parasite cross-talk was analyzed at five post-infection times 1, 6, 12 and 24 hours post-infection (hpi) and 8 days post-infection (dpi). A significant up-regulation of four interferon-induced proteins with tetratricopeptide repeats IFIT1, IFIT2, IFIT3 and MX1 was observed at 8 dpi suggesting a type 1 interferon (IFN) host response. Quantitative alteration of host proteins involved in biological functions such as signaling (STAT1, Ras) and reduction of the translation activity (EIF3) confirmed a host type 1 IFN response. Interestingly, the SILAC approach also allowed the detection of 148 *A. algerae* proteins during the kinetics of infection. Among these proteins many are involved in parasite proliferation, and an over-representation of putative secreted effectors proteins was observed. Finally our survey also suggests that *A. algerae* could use a transposable element as a lure strategy to escape the host innate immune system.

## Introduction

More than half of life in ecosystems is parasite and, as a life history strategy, parasitism has evolved more times than predation [1], [2]. A large part of the living organisms, at different times during the evolution of life and in each Kingdom opted and/or were under duress to invade other organisms, from their respective Kingdom or not, at extra- or intra- cellular levels [2]. This allowed them to reach an appropriate cellular niche (i.e. microhabitat), and gave them access to the nutrients needed for their growth and their survival. To achieve this, parasites need to hijack host cellular functions. For a large number of host-parasite associations, the arms race between partners has been ongoing for several hundred million years [2], [3], a struggle that has so far led to the selection of different parasite cellular lifestyles until the intracellular-obligate parasitic state. Although parasites exist in virtually every conceivable host niche, no parasite lifestyle is as specialized as obligate intracellular one. This parasite lifestyle influences access to nutrients, interactions with host cells signaling pathways and detection by parasite recognition systems. As such, intracellular life requires quite a repertoire of adaptations in order to ensure entry-exit from the cell, as well as to counter innate immune mechanisms and prevent clearance. The deciphering of this kind of host-parasite cross-talk at cellular and molecular levels is essential to the understanding of the key molecular strategies shared by obligate intracellular parasites in the hijacking of host cellular functions [2]–[5].

Microsporidia have been shown to cluster at the base of the fungal kingdom, as a sister group to chytrid pathogen *Rozella allomycis* [6]. They are all obligate intracellular parasites and 1,300 to 1,500 species in 187 genera were described that can infect a wide range of hosts from insects to mammals [7]. With some of the smallest eukaryotic genomes (from 2.3 to 24 Mb) and characterized by a strong host dependency the microsporidia phylum is therefore particularly interesting to study as a source of pioneer data on the host-intracellular parasite cross-talk. This strong host dependency is illustrated by an extensive gene loss and a genome compaction (i.e. the lack of and/or reduction in the number of genes coding for numerous metabolic pathways well conserved in eukaryotes, and also simplification of cellular processes such as transcription) [8]–[12]. Since their discovery in the 1850s as the causative agent of the silkworm disease (works of Balbiani and Pasteur) [10] which devastated the silk industry in Europe, many studies have been conducted on these intracellular parasites because of their major impact on animal farming, for instance in beekeeping (*Nosema ceranae* and *Nosema apis*), in sericulture (*Nosema bombycis*) and in aquaculture (*Loma salmonae* for salmonid or *Thelohania* spp. for shrimp). Microsporidia have also been considered as opportunistic parasites in human and listed as a public health threat since the AIDS pandemic. The Microsporidia had been added to the National Institute of Allergy and Infectious diseases priority parasite list (category B, Biological Diseases, Food and Waterborne Pathogens) [10]. *Anncaliia algerae*, was first isolated from *Anopheles stephensis* at the aquatic larval stage. It is one of the microsporidian species with the broadest known host range [13] and can infect both immuno-competent and immuno-compromised patients [13]. Furthermore, mosquitoes co-infected with *A. algerae* and *Plasmodium falciparum* exhibit reduced *P. falciparum* development, suggesting that *A. algerae* enforces a biological defense against the causative agent of malaria [14]. Finally, *A. algerae* is an appropriate parasite model to study host-intracellular parasite cross-talk because of its ability to grow *in vitro* within a large diversity of cells and temperatures [15], and of the availability of its complete genome sequence harboring only 2,075 protein encoding genes [16].

Many scientists are heavily betting on “omics” tools to decode cross-talk in host-parasite associations and, thus, to first understand parasite molecular strategies to bypass host defenses. However, although genomic tools can provide great insights in such quests, the execution of the genetic plan is carried out for a large part by the proteins activities [17] and that is why many proteomics studies in the last decade have aimed at elucidating the host-parasite interactions. The use of proteomics has been promoted by the development of new user-friendly tools such as free gel approaches [18], [19]. Quantitative metabolic labelling techniques such as SILAC (Stable Isotope Labelling by Amino acids in Cell culture) have frequently been coupled to Mass Spectrometry (MS) for acquisition of quantitative data on changes in protein abundance between cells or experimental conditions [18]. This powerful method was first used to identify and quantify relative differential changes in complex protein samples [20]. As a result, SILAC opens new opportunities for the elucidation of host-parasite cross-talk involved in the hijacking of host cellular functions by parasites [5], [21], and has been successfully used in similar studies on host-viruses interactions [21]. Our data deliver for the first time a temporal view of the host-parasite cross-talk during hijacking of host cellular functions in a minimal host-parasite interaction model. Key insights are the host interferon response against a microsporidia and the possible activation of parasite transposable elements (i.e. lure parasite strategy against host innate immune system).

## Material and Methods

### Workflow

**Figure 1:**
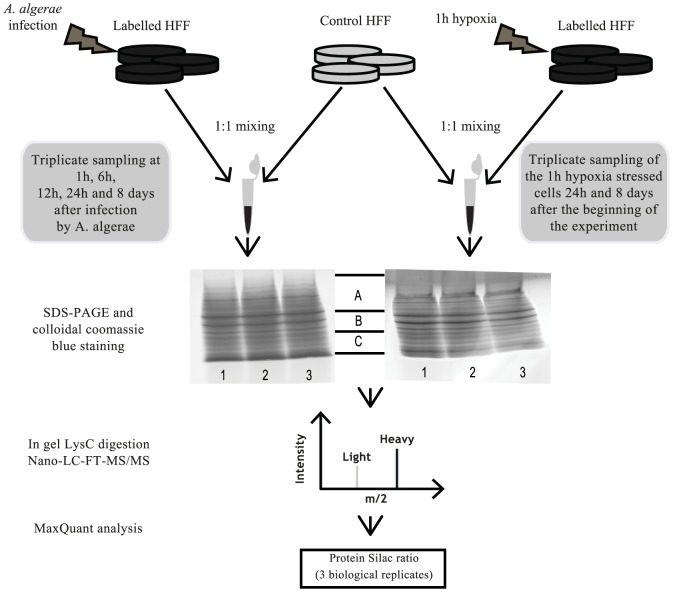
Workflow to decipher the molecular cross-talk between human cells and the obligate intracellular parasite *A. algerae* which is characterized by a strong host dependency. HFF cells labelled with ^13^C_6_^15^N_2_-lysine (dark gray) were submitted to either infection by *A. algerae* or hypoxic stress. For each time point of both kinetics the labeled cells were combined at 1∶1 ratio with unlabeled HFF cells (light gray) and proteins were extracted. For each sample a three biological replicate was made. The proteins samples were resolved on SDS-PAGE and total proteins lanes were cut in 3 regular pieces (A, B, C) and processed for in-gel digestion with endoproteinase LysC. LC-MS/MS analysis was then performed and mass spectra were analyzed with the MaxQuant software to achieve the relative protein quantification.

We investigated a minimal host-parasite interaction model (i.e. parasite with a strong host dependency) namely *A. algerae* while infecting human foreskin fibroblasts (HFF) to figure out the specific molecular cross-talk during the infection at two different time scales: early (1 hour post-infection (hpi), 6 hpi, 12 hpi and 24 hpi) and late (8 days post-infection (dpi)). In our experimental system, we observed meront stages 24 hpi, and after a proliferative step, mature spores are formed 3 dpi (figure S1). Finally, the majority of HFF cells are infected 8 dpi as shown in figure S1. In proteomics analysis several proteins have been shown to be modulated whatever the stress source [22]. In order to be able to spot and eliminate host proteins not being specifically modulated by the infection condition, we decided to compare with the host response when submitted to an abiotic stress (i.e. hypoxic) at two different cell ages (24 h and 8 d). Proteomics analyses were led by the labelling in cell culture of HFF with stable isotope (^13^C_6_^15^N_2_-lysine) amino acids before the infection (or hypoxic stress), then followed by a tandem mass spectrometry analysis of the proteins extracted at each time points (i.e. 1, 6, 12, 24 hpi and 8 dpi; 24 h and 8 d for the hypoxia). Using this experimental design, we were able to follow the specific kinetic response of both host and parasite proteomes at five post-infection times. The use of three biological replicates allowed a high sensitivity in the detection of weak relative changes in the abundance between different experimental conditions (Fig. 1).

### Host and parasite material

Human foreskin fibroblasts (HFF)(ATCC SCRC-1041) cells were grown in SILAC Dulbecco's modified MEM (DMEM, PAA) in a 5% CO_2_ incubator at 37°C. The *A. algerae* isolate used in this study was kindly provided by Pr. W.A. Maier (University of Sigmund-Freud, Bonn) and it is the same organism as in the original description of the parasite in the paper of Vavra and Undeen (1970) (reference ATCC PRA-339) [23].

### Culture medium, reagents and incorporation test

HFF cells were cultured as monolayers in DMEM supplemented with 10% heat inactivated dialyzed FBS (Invitrogen), 2 mM L-glutamine, arginine 84 mg/L, antibiotics (penicillin 100 U/ml-streptomycin 100 µg/ml, ampicillin 0.2 mg/ml) and fungicide (amphotericin B 0.25 µg/ml). For the SILAC experiments, cells were cultured in DMEM containing either ^13^C_6_^15^N_2_-lysine (heavy SILAC medium) or ^12^C_6_^13^N_2_-lysine (light SILAC medium, unlabeled lysine) at the concentration of 150 mg/L. After five cell population doublings on 75 cm^2^ culture flask, proteins were extracted from the heavy SILAC cells in 150 µl of Laemmli buffer (2% SDS, 10% glycerol, 5% 2-mercaptoethanol, 0.002% bromophenol blue and 0.0625 M TrisHCl pH 6.8), boiled for 15 min and centrifuged at 16,000×g at 4°C for 5 min. The protein concentrations of the cell lysates were determined with Bio-Rad Bradford Assay. The degree of incorporation of ^13^C_6_^15^N_2_-lysine was evaluated by mass spectrometry according to Ong and Mann work [24]. The heavy HFF cells were trypsinized and mixed with control cells at a 1∶1 ratio. The proteins were then extracted and quantified as described above. After separation by SDS-PAGE, total proteins were divided in 3 bands according to their molecular weight (Fig 1) and were processed for in-gel digestion with endoproteinase LysC. The peptides were analyzed by MS/MS. The heavy/light ratios were calculated for each protein.

### SILAC experimental procedure

HFF labelled cells at confluence in 75 cm^2^ flasks were treated with 1 ml of PBS, and exposed to two independent treatments (Fig. 1): (i) infected with 1×10^6^ fresh spores of *A. algerae* (biotic stress), or (ii) placed in hypoxia for 1 h (abiotic stress) before each sampling (24 h and 8 d after the beginning of the experiment). The hypoxia condition was achieved by filling the flasks of control HFF cells with N_2_, the flasks were then hermetically sealed with parafilm for one hour. For the infection, the spores of *A. algerae* were left 1 h in contact with host cells before 3 washing steps with fresh medium. The light (unlabeled) HFF cells (control) were also treated with 1 ml of PBS (control). Either heavy or light HFF cells were then washed 3 times with fresh complete medium and sustained in culture for 8 days. For each time points (1 h, 6 h, 12 h, 24 h and 8 d) used to decipher host responses, the labelled and unlabeled HFF cells were dissociated with 0.05% trypsin-EDTA (Gibco). They were washed three times in PBS and manually counted. For each treatment (infection or hypoxia) and for each time point, 3 replicates of heavy HFF cells were combined at 1∶1 ratio with light HFF cells, then centrifuged at 100 xg at 4°C for 5 min. The pellets were immediately frozen in liquid nitrogen and kept at −80°C until the end of the sampling procedure. Afterwards, proteins were extracted and quantified as described above.

### Mass spectrometry data acquisition and processing

All time points of the *A. algerae* infection kinetics (1 hpi, 6 hpi, 12 hpi, 24 hpi and 8 dpi) and the two of the hypoxia samples (24 h and 8 d) were resolved on 12%SDS PAGE using the protean II xi cell system (Bio-Rad laboratories, Marnes-La-Coquette, France). Gels were stained with PAGE-Blue protein staining solution (Fermentas Vilnius, Lithuania) and scanned using a computer-assisted densitometer (EPSON Perfection V750PRO). Gel lanes were cut in 3 regular pieces and destained with two washes in 50% acetonitrile/50 mM triethylammonium bicarbonate. After protein reduction (10 mM dithiothreitol at 50°C for 1 h), and alkylation (55 mM iodoacetamide at room temperature for 30 min), proteins were processed for in-gel digestion over night at 25°C using LysC (2.2 µg/band, Wako, Osaka, Japan). Digested products were extracted with 50% acetonitrile/50 mM triethylammonium bicarbonate and then 5% formic acid. Peptide solutions were dehydrated in a vacuum centrifuge. The generated peptides were analyzed online by nano flowHPLC-nanoelectrospray ionization using an LTQ-Xl Orbitrap mass spectrometer (Thermo Fisher Scientific) coupled to an Ultimate 3000 HPLC (Dionex, Thermo Fisher Scientific). Desalting and pre-concentration of samples were performed on-line on a Pepmap pre-column (0.3 mm×10 mm, Dionex). A gradient consisting of 0–40% B in A for 60 min, followed by 80% B/20% A for 15 min (A = 0.1% formic acid, 2% acetonitrile in water; B = 0.1% formic acid in acetonitrile) at 300 nL/min was used to elute peptides from the capillary reverse-phase column (0.075 mm×150 mm, Pepmap, Dionex). Eluted peptides were electrosprayed online at a voltage of 2.2 kV into an LTQ Orbitrap mass spectrometer. A cycle of one full-scan mass spectrum (400–2000 m/z) at a resolution of 60,000 (at 400 m/z) in the orbitrap, followed by 5 data-dependent MS/MS spectra (LTQ) was repeated continuously throughout the nanoLC separation.

Raw data analysis was performed using the MaxQuant software (V.1.3.0.5) [25]. Retention time-dependent mass recalibration was applied with the aid of a first search implemented in the Andromeda software [26] and peak lists were searched against the UniProt human database (release2013_04; http://www.uniprot.org) for the host response analysis and a local *A. algerae* database (supplementary data) for the parasite proteins detection, 255 frequently observed contaminants as well as reversed sequences of all entries. The following settings were applied: spectra were searched with a mass tolerance of 7 ppm (MS) and 0.5Th (MS/MS). Enzyme specificity was set to LysC. Up to two missed cleavages were allowed and only peptides with at least six amino acids in length were considered. Oxidation on methionine was set as a variable modification. Peptides identifications were accepted based on their false discovery rate (<1%). Accepted peptide sequences were subsequently assembled by MaxQuant into proteins, to achieve a false discovery rate of 1% at the protein level. Relative protein quantifications in samples to be compared were performed based on the median SILAC ratios, using MaxQuant with standard settings. Significance thresholds were calculated by using Perseus (www.maxquant.org) based on significance A with a p-value of 0.01 for normalized peptide ratios. Graphical representations were generated using the R statistical environment (V.3.0.1)[27].

### Bioinformatics analysis

A biological process Gene Ontology analysis was performed for each kinetic time points of the host. Differentially expressed proteins of the host at each time point were annotated with their Gene Ontology Biological Process terms [28] (Table S1). The annotation of the parasites proteome was conducted using Blast2Go (V.2.7.1)[29] with a BlastP algorithm using an e-value of 0.01 followed by an InterproScan step against the entire databases. The GO analysis was then finalized by merging the biological processes terms obtained after these two steps. The prediction of signal peptide cleavage sites was performed using the SignalP program, version 4.1 [30] set with the default parameters for eukaryotes.

## Results

### Host and parasite monitored proteins and incorporation rate of ^13^C_6_^15^N_2_-lysine

**Figure 2:**
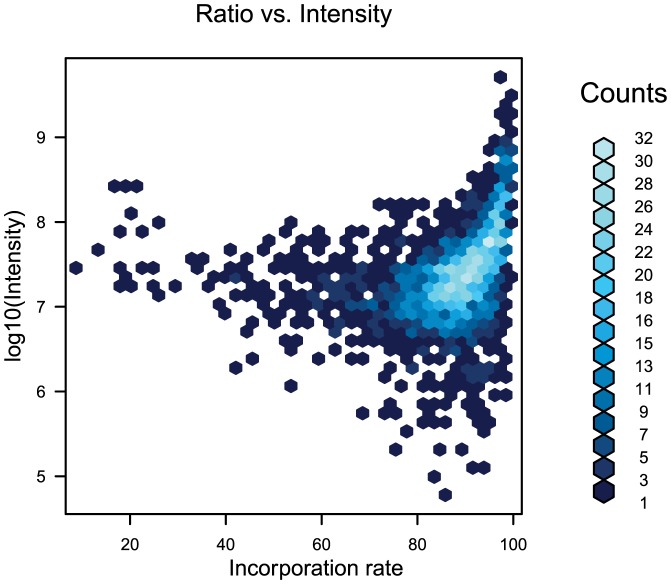
Incorporation rate of stable isotope ^13^C_6_^15^N_2_-lysine by HFF cells after five cell population doublings. Each hexagon is colored according to the number of proteins in its area of incorporation and intensity. Most of proteins present an incorporation rate above 90%, which is efficient for a good quantification of the protein changes in abundance for both infection and hypoxia treatments.

Heavy/light ratios were calculated for each detected protein. The median of the incorporation rate for each protein was above 90% for the HFF cells, which is efficient for a good quantification of the host protein changes in abundance (Fig. 2). A total of 1,190 proteins were identified in the three biological replicates during the cross-talk between human cells and the microsporidian parasite: 1,041 for were quantified and corresponded to proteins from HFF cells. The 148 other proteins were from *A. algerae* and were probably detected because they were the most abundant parasite proteins (Tables S2, S3). Analysis of the host proteome response to the hypoxic treatment revealed a total of 1,194 human proteins in the three biological replicates during the kinetics (Table S4). The correlation of the heavy/light protein abundance ratios showed a high reproducibility of protein quantification in the three biological replicates for each time points and for both stress conditions (i.e. parasite infection and hypoxia; Table S5).

### Host proteome modulation

**Figure 3:**
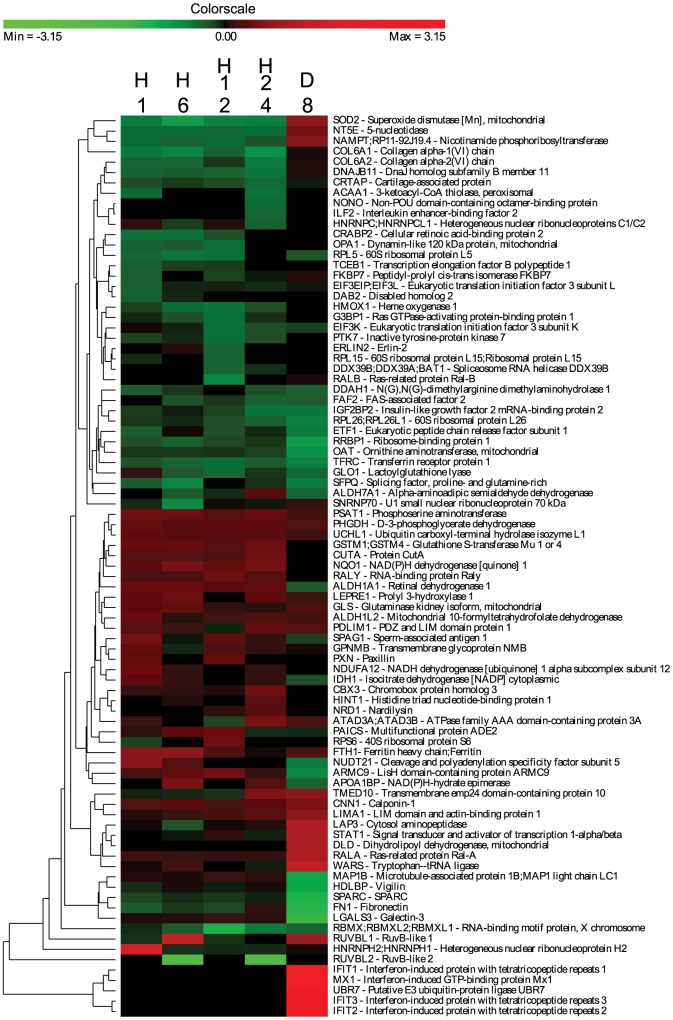
Clusters of the host proteins significantly modulated during the kinetics of infection. HFF cells at confluence were infected by 1×106 spores of A. algerae and samples were taken 1 hour post infection (H1), 6 hour post infection (H6), 12 hour post infection (H12), 24 hour post infection (H24) and 8 day post infection (D8). Log2 Protein ratio were measured using the SILAC workflow and were relative to the uninfected cells. Genes were selected for this analysis if their expression level differed significantly from the control for at least one time point. The color scale ranges from saturated green for log ratios −3.15 and below to saturated red for log ratios 3.15 and above. Each gene is represented by a single row of colored boxes and each time point is represented by a single column.

**Figure 4:**
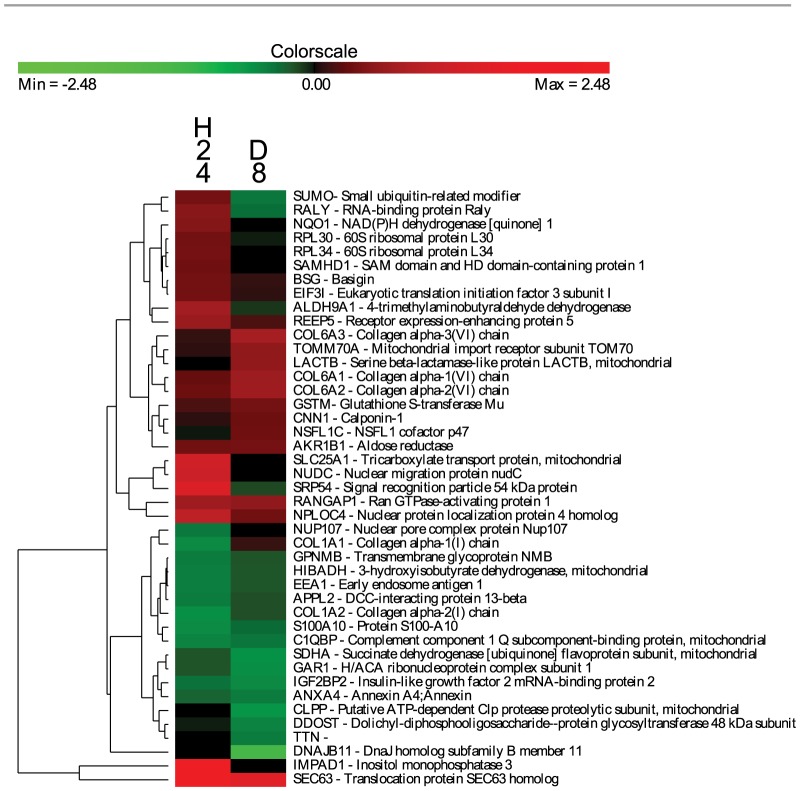
Clusters of the host proteins significantly modulated during the hypoxia treatment. HFF cells at confluence were submitted to an abiotic stress (i.e. hypoxic) at two different cell ages 24 hours (H24) and 8 days (D8). Proteins expression levels were clustered by using methods described here before in Fig S1.

During the HFF cells-*A. algerae* cross-talk, 87 proteins of HFF cells significantly varied in expression ratio in at least one of the five time points (Fig. 3). Forty-three HFF proteins were differentially expressed after the hypoxic stress (Fig. 4) while only nine proteins were shared between infection and hypoxic treatments. These common proteins corresponded to three proteins of the cytoskeleton and the extracellular matrix (COL6A1, COL6A2, CNN1), two proteins involved in detoxification process (GSTM, NQO1), two involved in transcription/translation regulation (RALY, IGF2BP2), one HSP related protein (DNAJB11) and one protein of unknown function (GPNMB). These nine proteins were discarded for further interpretation of the kinetics of infection by *A. algerae*. For each kinetic time point, differentially expressed host proteins were annotated with GO Biological Process terms [28]. The percentage of dysregulated proteins annotated to each GO term is reported in Table S1.

### Parasite proteome

Among the 148 parasite proteins detected during the HFF cells-*A. algerae* temporal cross-talk (Table S3). Most of these proteins were only detected for the 8 dpi time point, except for three: the 26S proteasome regulatory subunit 10 (detected at 6 hpi, 12 hpi, 24 hpi and 8 dpi), the endoplasmic reticulum membrane protein (detected at 6 hpi) and the glutamine amidotransferase (detected at 1 hpi, 24 hpi and 8 dpi). From the 148 *A. algerae* proteins, 20 showed no significant similarity with proteins in the RefSeq non-redundant database (nr) [31], even with an e-value threshold of 0.01. This suggests that these sequences are quite divergent from other known organism sequences. The SignalP program predicted the presence of a N-terminal signal peptide for 18 *A. algerae* proteins (Table S3). Interestingly Fisher's exact test confirms the over-representation of predicted secreted proteins among the 148 identified proteins (p-value = 3.467×10^−4^) when compared to the predicted secreted proteins from the whole *A. algerae* proteome.

## Discussion

To date, the available proteomics data on host-parasite interactions are mainly about the host's response to the parasite [4], [5], [32], [33]. Both the low parasite size of intracellular parasites and the low amount of parasite proteins compared to their host cells can explain the difficulty to extract enough parasite proteins from host cells suitable for detection during the kinetics of the host-parasite cross-talk. For the first time the host-parasite cross-talk was investigating in the context of an eukaryotic intracellular parasite using the highly sensitive and quantitative SILAC approach. We identified 148 parasite proteins most of them being detected at 8 dpi. The parasite proteins with known functions were mostly involved in fundamental biological processes and reflect the successfully multiplication of the parasite inside the host. Regarding the host dialogue during the kinetics of *A. algerae* infection, since our model was produced in cell culture, the host cells response to *A. algerae* mainly involved in the innate immune response split in two parts: first the oxidative stress and then the type I interferon response.

### Antioxidant proteins involved in the protection against the oxidative stress induced by the infection

Glutathione-S-Transferase (GST), Superoxide Dismutase 2 (SOD2), and ferritin were strongly modulated in *A. algerae*-infected HFF cells. GST is overexpressed and is part of the classical response to oxidative stress due to the production of reactive oxygen species (ROS) to counter an infection. This had already been observed following an infection of *Aedes aegypti* by the microsporidian species *Vavraia culicis* and *Edhazardia aedis* [34], [35]. Conversely, the mitochondrial SOD2 protein was strongly downregulated until 24 hpi, hence questioning its role in the antioxidant system. However, this modulation could also be caused by a strong disturbance of the host mitochondria consecutive to cell infection, as it is known that microsporidia are highly dependent on their host pertaining to their ATP supply [8], [10]. A SILAC experiment focusing on mitochondria fraction of infected cells (vs. healthy cells) could be helpful to understand how microsporidia can hijack the host mitochondria metabolism. Another well-known ROS implicated in the innate response is the inducible nitric oxide (iNOS). This ROS is produced by the nitric oxide synthase (NOS) through the L-arginine and L-ornithine pathways which are tightly regulated by different enzymes including the ornithine aminotransferase (OAT) [36]. In our experiment, the OAT but also the N(G),N(G)-dimethylarginine dimethylaminohydrolase 1 (DDAH1, a NOS inducer) [37] proteins were less abundant during the infection suggesting that the NOS regulation is altered by the infection as it has been observed in several parasitic infections [34], [35]. This modulation could be linked to the strong upregulation of the ubiquitin carboxyl-terminal hydrolase isozyme L1 (UCHL1) observed in our experiment. Indeed, it is known that the NOS synthesis is regulated through the activation of the extracellular signal-regulated protein kinase (ERK) and the UCHL1 protein has been shown to drastically decrease the activation of ERK [38], arguing for an inactivation of the NOS system in *A. algerae*-infected HFF cells.

### Interferon response specific to *A. algerae*

**Figure 5:**
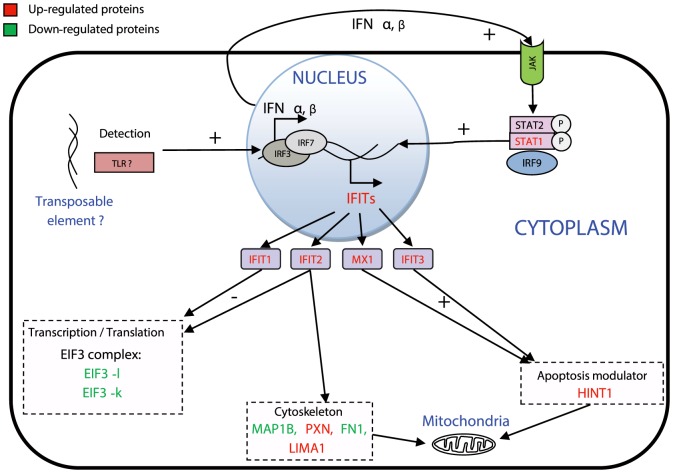
Schematic illustration of the proposed IFN host response against infection with *A. algerae*. Proteins that have been shown to be upregulated during the experiment are indicated in red, the downregulated ones are in green. The dsDNA produced by the TE is recognized by the Toll-like receptor TLR9 which senses CpG DNA. This leads to the activation of IRF3 and IRF7 by phosphorylation, which can bind host genome to stimulate IFN expression. The secreted IFN would then activate the JAK-STAT signaling pathway. The phosphorylated STAT1, STAT2, and IRF9 form the ISGF3 complex, which is translocated into the host nucleus, and thus stimulates IFIT family genes expression. IFIT1 and IFIT2 directly bind eIF3 and suppress transcription. IFIT2 interacts with MITA (Mediator of IRF3 activation), and induces apoptosis via the mitochondrial pathway that is induced by the innate immune response. IFIT2 can also interact with microtubules and could be responsible of cytoskeleton reorganization.

In *A. algerae*-infected cells, the IFIT1 (IFIT for Interferon-induced protein with tetratricopeptide repeats), IFIT2, IFIT3 and the interferon-inducible MX1 proteins were dramatically upregulated after 8 dpi, with a log2 ratio upper to 2.5 (Fig. 3, Fig. 5). These four proteins are known to be induced by type I and type III interferons, especially IFN-α/β [39]. Interferons (IFNs) are a family of proteins secreted by host cells in response to infection by various intracellular pathogens such as viruses, bacteria, fungi or protozoa, and involved in the innate immunity. This mechanism is well documented in the case of viral infections, but few studies have described type I interferon production in response to intracellular parasites, as it has been the case for *P. falciparum* [40] or *Listeria monocytogenes* [41]. Once IFN is produced and secreted by the cell, it acts in an autocrine and paracrine loop to stimulate its receptor on both the infected and neighbouring cells (Fig. 5). To explain the late detection of IFN response, the critical mass of infected cells required to detect this auto activation loop might need more than 48 h of infection in our experimental conditions. Proteins involved in cell signaling were also detected as modulated during the infection, especially the proto-oncogene Ras and proteins involved in the JAK-STAT pathway. This pathway has already been shown to be modulated in the case of mice infection by *L. monocytogenes* as a direct consequence of the IFIT-induced system [39]. Following this first step, the extracellular-produced IFN-β can bind to the Janus Kinase receptor (JAK) leading to the activation of STAT1 (which is upregulated in our experiment) and STAT2. These two proteins form a complex with IRF9 (interferon regulatory factor 9), which translocate into the nucleus and promotes the expression of the IFIT family genes. As previously mentioned, proteins involved in Ras signal transduction were modulated by the infection, suggesting an activation of the pathway. Moreover, Ras signaling impacts numerous functions in the cell including actin cytoskeletal integrity, proliferation, differentiation, cell adhesion, apoptosis, and cell migration.

Among the different altered functions which are linked to the interferon response, a cluster of proteins responsible for transcription and translation is shown to be downregulated at any time of the infection (consistent with a Type I interferon response). Indeed, IFIT1 and IFIT2 are known to directly bind to the EIF3 complex resulting in a suppression of more than 60% of translation in cells [42]. Interestingly, in our experiment, EIF3-l and EIF3-k, two components of the EIF3 complex are downregulated until 6 hpi. We also observed that the 60 S ribosomal protein is strongly down-regulated during the experiment confirming a decrease in protein translation activity.

Nine proteins associated to the cytoskeleton were modulated during the infection suggesting a reorganization of the host cell. Such a cell structure reorganization is well known in the case of red blood cells infected by *P. falciparum* [43]. According to its obligatory parasitic nature, *A. algerae* is highly dependent of its host especially for energy supply [10]. *A. algerae* may be tightly associated with the host mitochondria and a relocalization of mitochondria around the parasitophorous vacuole has been already observed after infection of mammal cells by the microsporidian species *Encephalitozoon cuniculi* [44]. Interestingly, one of our modulated proteins, the microtubule-associated protein 1B (MAP1B), is responsible for the mitochondria transport through the microtubule web [45]. As IFIT2 can interact and modulate the microtubule network [46] we hypothesized that IFIT2 could be implicated in cell reorganization during *A. algerae* infection.

Apoptosis is a classical response to intracellular pathogens especially after viral infection; this phenomenon could also be induced through the IFN response. IFN-induced proteins have the ability to block the cell cycle, promote apoptosis or lead to cell proliferation [47]–[49]. In our experiment, the effects of infection on the apoptosis process remain unclear given that no direct effector of apoptosis has been detected. Nevertheless, IFIT3 and HINT1, which are tightly upregulated in our experiment, have been shown to induce apoptosis through the mitochondria pathway [48] and an upregulation of P53 and Bak transcription, respectively [50]. Our results therefore suggest an activation of the apoptotic signalization, however, the direct detection of apoptotic processes would be necessary to conclude on this aspect. Despite the oxidative stress and the interferon response are induced during the infection of HFF cells by *A. algerae*, the parasite succeeds in its infection process, meaning that these two arms of the immune system are inefficient or not sufficient enough to counter *A. algerae*.

### Predictive host-parasite crosstalk

Among the parasite proteins, we found that 12% are predicted to be secreted. This is intriguing since secreted proteins represent only 5% of the complete *A. algerae* predicted proteome. The over-representation of these proteins can have a biological significance as they can be found at the host-parasite interface and may interfere directly with host cellular components. However we cannot exclude that this could be the result of an extraction bias since these secreted proteins are more easily extracted. Unfortunately, the functions of most of these proteins are unknown, and further investigation is still needed to demonstrate their role as potential parasite effectors. To protect itself against host defenses (ROS), the parasite may have an antioxidant system since we detected proteins involved in ROS detoxification (ferritin, thioreductase and superoxide dismutase) (Table S3).

Interestingly, among the parasite proteins, a transposable element was detected (TE)(POL polyprotein), supporting a recent study showing that *A. algerae* genome harbors numerous TEs [16]. Usually the activity of these elements is under regulation of the host, via pre- and post-transcriptional mechanisms [51] which thus makes their proteins difficult to detect. We found one specific microsporidian regulation signal within the putative promoter of this TE, i.e. a GGG-motif before the start codon [16], suggesting a domestication of the TE by the microsporidia, that may have provided an advantage in the evolutive history of *A. algerae*. The domestication of TEs in order to fulfill particular functions for the host organism has been a recurrent event throughout evolution and concerns any type of TEs [52], [53]. Host organisms have thus repeatedly recruited both the regulatory and the coding sequences of TEs. These co-optations can be very ancient like the telomere maintenance by TART and HeT-A retrotransposons in *Drosophila* [54], the programmed genome rearrangements of *Paramecium* by a DNA transposon [55]. Ancient co-optation were also showed for the syncitin proteins, derived from the envelope gene of an endogenous retrovirus, which are implied in the placenta formation in mammals [56], or the RAG1 and RAG2 proteins promoting the V(D)J recombination to generate the diversity of immunoglobulins in vertebrates that both derived from a DNA transposon [57]. In the last two cases, the structure of the TE is no more recognizable and the recruited TE sequence has become a new host gene. More recent cases of domestication have also been described like the ENS1/ERNI protein which is specifically produced in the embryonic stem cells of chicken and controls the timing of the neural plate emergence, which function is probably restricted to the galliform species and which structure still mainly corresponds to that of a LTR retrotransposon [58], [59]. The adaptive advantages of some TE insertions have also been shown to occur quite often in *Drosophila*, some of these events being particularly recent since they took place less than 10,000 years ago [60].

In the case of type 1 IFN response to *L. monocytogenes* infection [41], the detection seems to be mediated by the direct recognition of cytosolic bacterial dsDNA by Toll like receptor 9, which is activated by CpG DNA. Considering this, we hypothesize that this TE could be the cause of the interferon response activation by leading to DNA release, since this protein still has the ability to synthetize dsDNA from RNA with its reverse transcriptase/ribonuclease H (RT) activities. One possible assumption is that the gene coding the POL polyprotein could have been selected during the co-evolution of mammals and *A. algerae* permitting the parasite to invade and grow in mammal host. The interferon response could then be detrimental to the host as it has been observed for intracellular pathogenic bacteria [61], and this could make the case that the interferon response is a parasitic lure strategy against host innate immune system.

## Supporting Information

Figure S1 ***A. algerae*** **intracellular developmental stages in the cytoplasm of HFF cells during 8 days-kinetics of infection.** H: hours, D: days.
(JPG)

Table S1 **Biological process Gene Ontology analysis for each kinetics time points of the host response.** Differentially expressed proteins at each time point were annotated with GO Biological Process terms.
(XLSX)

Table S2 **Host proteins identified during the kinetics of infection and significance score for each time of the kinetics.**
(XLS)

Table S3 **Parasite proteins detected during the kinetics of infection.**
(XLSX)

Table S4 **Host proteins identified in hypoxia experiment and significance scores for each time of the kinetics.**
(XLSX)

Tables S5 **Correlation (Pearson's coefficient) of the heavy/light protein abundance ratios between the three biological replicates for each time points studied for both stress (i.e. parasite and hypoxia).**
(DOCX)
