# Supplementary figures and images for: Hijacking of Host Cellular Functions by an Intracellular Parasite, the Microsporidian Anncaliia algerae
Source: PLoS One. 2014 Jun 26;9(6):e100791. doi: 10.1371/journal.pone.0100791 (PMC4072689; doi:10.1371/journal.pone.0100791)

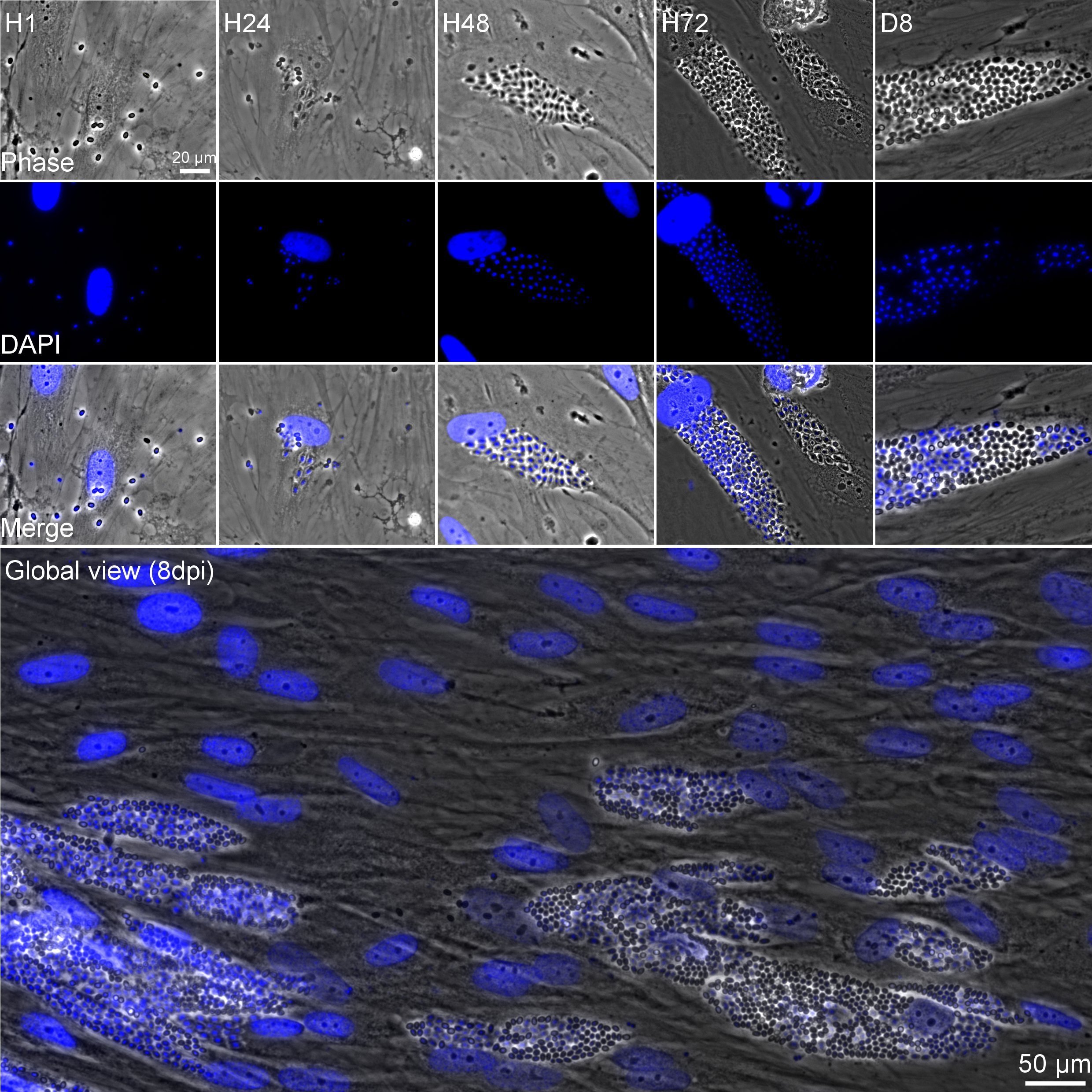

Supplement: Figure S1 — A. algerae intracellular developmental stages in the cytoplasm of HFF cells during 8 days-kinetics of infection. H: hours, D: days. (JPG) [file pone.0100791.s001.jpg]
