# Supplementary material for: Hijacking of Host Cellular Functions by an Intracellular Parasite, the Microsporidian Anncaliia algerae
Source: PLoS One. 2014 Jun 26;9(6):e100791. doi: 10.1371/journal.pone.0100791 (PMC4072689; doi:10.1371/journal.pone.0100791)
Supplement: Tables S5 — Correlation (Pearson's coefficient) of the heavy/light protein abundance ratios between the three biological replicates for each time points studied for both stress (i.e. parasite and hypoxia). (DOCX) [file pone.0100791.s006.docx]

**Table S5 : Correlation of the heavy/light protein abundance ratios between the three**

**biological replicates for each time points studied for both stress (i.e. parasite**

**and hypoxia).**

|  | **Infection** | | | | | **Hypoxia** | |
| --- | --- | --- | --- | --- | --- | --- | --- |
| **Comparison of replicates** | **H0** | **H6** | **H12** | **H24** | **D8** | **H24** | **D8** |
| 1-2 | 0.80 | 0.72 | 0.85 | 0.90 | 0.90 | 0.52 | 0.75 |
| 1-3 | 0.81 | 0.84 | 0.87 | 0.79 | 0.97 | 0.71 | 0.77 |
| 2-3 | 0.87 | 0.75 | 0.83 | 0.79 | 0.88 | 0.48 | 0.75 |
